# Association of Received Intergenerational Support with Subjective Well-Being among Elderly: The Mediating Role of Optimism and Sex Differences

**DOI:** 10.3390/ijerph19137614

**Published:** 2022-06-22

**Authors:** Zixin Pan, Ji-Kang Chen

**Affiliations:** Department of Social Work, Chinese University of Hong Kong, Hong Kong, China; jkchen@swk.cuhk.edu.hk

**Keywords:** intergenerational support, subjective well-being, optimism, sex differences

## Abstract

Even though an extensive body of previous research has examined the association between received intergenerational support and the well-being outcomes of older adults in a wide variety of contexts, few studies have been conducted to explore the impacts of intergenerational support on elders’ subjective well-being, especially the intermediary mechanisms in this process. The purpose of this study is to fill this gap by exploring the mediating role of optimism in the association between received intergenerational support and subjective well-being among the elderly in China, as well as the sex differences that exist between males and females. The findings show that the intergenerational support received from adult children is positively related to subjective well-being and that this relationship is partly mediated by optimism. Meanwhile, no significant sex difference was found in the interrelations between intergenerational support, optimism, and subjective well-being.

## 1. Introduction

Individuals’ subjective well-being is defined as their overall and comprehensive evaluation of the quality of life according to their own standards and it plays a central role in healthy aging [1,2]. Several studies have found that social support from family members, friends, and neighbors significantly improves the subjective well-being of elders [3,4]. Moreover, support from family members, especially adult children, has a greater impact on health-promoting behaviors and healthy aging outcomes than other support from friends or neighbors [5,6,7]. Received intergeneration support, defined as a type of familial social support received by the elderly from their adult children [8,9], has been identified as having three types in the existing literature: financial, instrumental, and emotional support [10]. Until now, a wide variety of theoretical perspectives and studies have suggested that receiving intergenerational support influences elders’ well-being-related outcomes [9,11,12,13].

However, the empirical studies on the association between received intergenerational support and the well-being outcomes of elders are inconsistent. On the one hand, some scholars have found that older parents who received intergenerational support from adult children are likely to have better health-related outcomes [5,14]. On the other hand, other studies have suggested that receiving intergenerational support, especially financial and instrumental support, may compromise elders’ well-being by inducing personal dependency and family strain caused by the reallocation of scarce resources [15,16]. Furthermore, some researchers have failed to identify any significant correlation between intergenerational support and the well-being outcomes of the elderly [17]. These contradictory findings further spur the investigation of the potential psycho-social mechanisms between received intergenerational support and the health-related outcomes of older adults [18,19]. Moreover, up until now, little research has been conducted on the effects of intergenerational support on elders’ subjective well-being, and there has been far less discussion on the potential intermediary variables underlying this association [20].

Scholars believe that personality traits, including core self-evaluation, are closely correlated with social support and well-being outcomes [20,21,22]. Optimism, as a type of personality characteristic, is defined as the generalized tendency for individuals to expect positive results even when they encounter obstacles [23]. According to social-cognitive theory, personal expectations, such as optimism, have a significantly influential effect on behavior, goals, and functioning among humans [24]. Thus, existing studies have examined the joint contribution of social support and optimism to health outcomes among a variety of other populations [25]. For example, studies found that optimism mediates the link between social support and well-being outcomes among the general adult population [26]; university student-athletes [27]; and preterm mothers [28]. However, whether this mediating effect is still valid among elderly populations remains unclear. Unlike other groups of people, older adults who experience a decline in their social network in the aging process primarily receive support and care from their family, especially their adult children, in most developing countries [29], highlighting the importance of intergenerational support in contributing to subjective well-being in old age. It further redirects our attention to the interactive effects of intergenerational support, in particular from adult children, and optimism on elders’ subjective well-being.

In addition, previous studies have indicated that sex differences have been found in rates of receiving support from adult children [30,31], subjective wellbeing [32,33], and optimism [34]. Furthermore, sex may also confound the relationship between support-relevant interactions and emotional well-being outcomes. In line with the differential vulnerability theory, women may be more psychologically susceptible to social support because they are generally more relationship-oriented and more actively involved in various forms of support as opposed to men [35]. Although some researchers have investigated sex differences in the association of received intergenerational support with subjective outcomes [35,36], we know less about such sex differences when introducing intermediate variables (such as optimism). To the best of our knowledge, no studies have examined whether optimism as the mediator in the relationship between received intergenerational support and subjective well-being varies across sex groups.

Therefore, by adopting nationally representative data from the Chinese General Social Survey (CGSS) of 2017, this study examined the direct effects of receiving intergenerational support on older parents’ subjective well-being, as well as their indirect linkage through older parents’ sense of optimism. Furthermore, our study also investigated whether any sex differences exist in the abovementioned pathways. Utilizing the abovementioned literature and theories (e.g., social-cognitive perspectives), this study proposes the following hypotheses. First, we expect that the level of intergenerational support received and elderly subjective well-being would be positively related. Second, we expect that optimism would serve as a mediator between received intergenerational support and subjective well-being. Last, we expect that significant differences between male and female elderly in the proposed theoretical model would be as predicted.

## 2. Method

### 2.1. Participants

The data in this study are based on the Chinese General Social Survey (CGSS) of 2017, a nationally large-scale social survey project held by the National Survey Research Center at Renmin University of China. By collecting data on the Chinese people and various aspects of Chinese society systematically, this survey is intended to summarize the long-term trends of social change in China [37]. CGSS 2017 collected data from 125 counties in 28 provinces in mainland China by employing a survey, including questions on family relationships and individual well-being. The participants in this study were older adults over the age of 60 who have at least one living adult child. After removing missing values and invalid variables from the total sample, the research found that a total of 1287 valid samples (including 599 males and 688 females) could be obtained based on the issues discussed in the study.

### 2.2. Measurements

#### 2.2.1. Intergenerational Support

The questionnaire asked participants to identify a child with whom they have the most frequent contact either face-to-face, by phone, by mail, or on the Internet (aged 18 or older). Then, we assessed three types of intergenerational support provided by the focal child in terms of financial, instrumental, and emotional support using the following questions: “How frequently did your focal child do each of the following things for you during the past 12 months?: (a) provide financial support, (b) take care of household chores or child or other family members, and (c) listen to your ideas and share their feelings.” All items were rated by the participants on a five-point Likert scale ranging from 1 to 5, indicating whether the child did not do it at all, rarely, sometimes, often, or very frequently, respectively. Higher scores indicated that the elderly parents received more intergenerational support. Previous literature has widely applied this scale to the Chinese elderly [18]. In the present study, the Cronbach alpha coefficient was 0.67.

#### 2.2.2. Optimism

Participants’ optimism was measured by the subscale of The Chinese Revised Life Orientation Test (CLOT-R) [38]. The *Life Orientation Test* (LOT) was developed by Scheier and Carver to assess individual differences in generalized optimism versus pessimism [23]. The revised version, developed by Scheier, Carver, and Bridges [39], has been found to be a reliable and valid measure among Chinese older adults [40]. The optimism subscale contains 3 items: “In uncertain times, I usually expect the best.”, “I’m always optimistic about my future.”, “Overall, I expect more good things to happen to me than bad.” Respondents rated each item on a five-point Likert scale: 1 = strongly disagree, 2 = disagree, 3 = neutral, 4 = agree, and 5 = strongly agree. Higher total scores indicated higher levels of dispositional optimism. The scale has been used to examine the elderly’s optimism in China with good reliability and validity [41]. In this study, the Cronbach alpha was 0.68.

#### 2.2.3. Subjective Well-Being

Subjective well-being was assessed through the 36th question from CGSS 2017. The question was “Overall, do you think your life is happy?” Possible answers were “1 = very unhappy”, “2 = unhappy”, “3 = not possible to say happy or unhappy”, “4 = happy”, and “5 = very happy”. A higher score indicated a higher level of subjective well-being.

### 2.3. Analytical Approach

First, we analyzed the descriptive statistics and bivariate correlation for the variables in this study. Further, structural equation modeling (SEM) using maximum likelihood estimation was carried out using AMOS 25.0 [42]. Confirmatory factor analysis (CFA) was conducted to confirm the validity of each measurement used in this study. Moreover, in order to examine the mediation effect of optimism on the association between received intergenerational support and the subjective well-being of elderly parents, bootstrapping (*n* = 2000 bootstrap samples) was employed [43]. In addition, the SEM was analyzed in more detail to examine whether there was a difference between male and female elderly in the theoretical model.

## 3. Results

### 3.1. Descriptive Results

In Table 1, the means and standard deviations of the variables are displayed for each sex. Table 2 displays the correlations between the variables. Receiving instrumental and emotional support is significantly related to optimism and subjective well-being. With more received instrumental and emotional support, elders are more likely to be optimistic and have a higher level of happiness. Additionally, optimism is significantly linked with subjective well-being, which suggests optimists tend to be happier.

### 3.2. Overall Model

**Figure 1:**
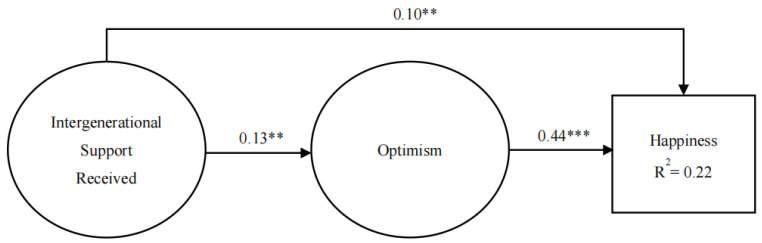
Overall model. *Note*. *** *p* < 0.001; ** *p* < 0.01.

The results of the confirmatory factor analysis showed that all factor loadings in the measurement model are statistically significant at the level of 0.001, ranging from 0.51 to 0.79. Furthermore, the structural model indicated a good model fit to the data, Chi-square = 60.31, DF = 12, NFI = 0.96, IFI = 0.97, CFI = 0.97, and RMSEA = 0.06. The results of the structural model are shown in Figure 1. First, intergenerational support has a significant direct association with happiness. With received intergenerational support increasing by 1 standard deviation, happiness increases by 0.10 standard deviations. In addition, intergenerational support is significantly associated with optimism (β = 0.13), and optimism significantly relates to happiness (β = 0.44). Furthermore, we employed a bootstrapping strategy with the 2000 samples that were created using random sampling from the original dataset to test the indirect effect of received intergenerational support on subjective well-being through optimism as the mediator. The results revealed that the indirect effect of intergenerational support on elderly parents’ subjective well-being through optimism was 0.06 (SE = 0.02, CI = [0.02, 0.10], *p* < 0.05). This supports the theory that receiving intergenerational support had a significant indirect effect on subjective well-being through optimism.

### 3.3. Sex Comparison

**Figure 2:**
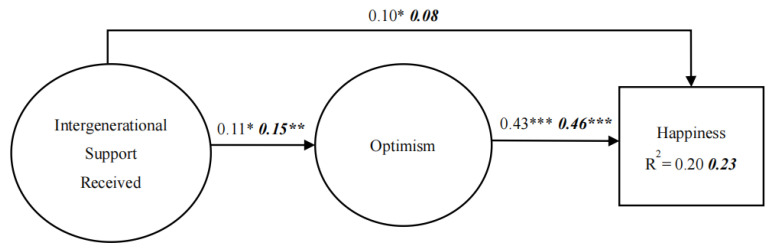
Sex comparison. *Note*. The coefficients in regular print and those in bold italics represent the results for male and female samples, respectively.*** *p* < 0.001; ** *p* < 0.01; * *p* < 0.05.

In this multi-group analysis, we compared the unconstrained model and the constrained model to examine whether sex differences exist in the theoretical model. For the constrained model, we set the factor loadings and structural paths as equal. Based on the results, the model fit indices were considered acceptable: Chi-square = 90.73, DF = 31, NFI = 0.94, IFI = 0.96, CFI = 0.96, and RMSEA = 0.04. When comparing the constrained and unconstrained models, we did not find significant Chi-square differences (CMIN = 6.24, *p* > 0.05), suggesting that male and female groups have no significant differences in the theoretical model. As shown in Figure 2, the results indicated that the regression coefficients for each path and the explained variance of the dependent variable for male and female elders were similar in the model.

## 4. Discussion

The objective of this study was to evaluate the direct effects of receiving intergenerational support on elders’ subjective well-being, as well as the indirect effects through elders’ optimism. In addition, sex differences in the pathway were examined. First, the findings indicated that receiving intergenerational support is significantly associated with the subjective well-being of elderly people. Second, elders’ sense of optimism plays a mediating role between the received intergenerational support and subjective well-being. Furthermore, no sex difference exists in the theoretical model.

### 4.1. Intergenerational Support and Subjective Well-Being

The findings show that elderly parents who receive more intergenerational support from adult children are likely to have a higher level of happiness. This is consistent with many previous studies on intergenerational solidarity that found upward intergenerational support from younger generations to older generations can lead to an improvement in subjective well-being [3,14,20]. For example, one study from China conducted by Tian [20] concluded that children’s support and assistance for their parents can increase the elderly’s subjective well-being. The relationship between accepting intergenerational support and subjective well-being can be explained by Bowling’s point of view that social support can maintain the organism by promoting adaptive behavior when under stress or other health threats [44]. Intergenerational support, such as providing elders with financial assistance, helping them with housework, and listening to their ideas and sharing their feelings, can buffer the stressful events in the aging process (e.g., financial distress after retirement, declining physical and physiological functions, and lack of social connections), thereby improving their emotional well-being [8,45]. In addition, according to Cicirelli [46], the higher degree of attachment between parents and adult children tends to correlate with better health and well-being for aging parents. Therefore, researchers contend that older people may become closer to their adult children and more satisfied with their intergenerational relationships by receiving intergenerational support, which may increase the perceived social support and benefit the elder’s mental health [19,47].

Concerning the impacts of the different types of intergenerational support (i.e., financial, instrumental, and emotional), it can be concluded that not all forms of intergenerational support affected the elderly parents’ subjective well-being in the same way. As shown in the present study, emotional support from adult children plays the most significant role in enhancing the subjective well-being of the elderly with a coefficient of 0.13. Additionally, a significant correlation exists between instrumental support and happiness (coefficient = 0.11). Therefore, by receiving more emotional/instrumental support from adult children, older parents tend to have a higher level of subjective well-being. However, receiving financial support is not significantly associated with the subjective well-being of elderly parents. The results are consistent with the argument that the psychological well-being of elderly people is more improved by emotional support than by financial or instrumental support [8].

### 4.2. Mediating Role of Optimism

Optimism refers to elders’ positive expectancies for the future when they encounter problems and difficulties in life [48]. As a result of the current study, our hypothesis regarding the mediating effect of seniors’ optimistic beliefs on the association between received intergenerational support and subjective well-being has been supported. The elderly who receive support from their adult children are likely to have a higher level of optimism, which in turn, results in increased subjective well-being. On the one hand, the results echo previous studies that show that intergenerational support is positively correlated with optimism. Although most of the previous studies proposed that individuals’ optimism helps increase their social support [49,50,51], the emerging literature has proposed a converse causal relationship between social support and individuals’ optimism [27,28]. For example, as suggested by Kestler-Peleg and Lavenda [28], optimism that reflects positive expectations of the future is derived from a positive appraisal of the individual–environment interaction in the present or a strong belief that things are going to be better in the future. To maintain such optimistic beliefs, people can draw on positive evaluations of the social context in which they are supported and can cope successfully with the challenges of life [52]. Therefore, elders receiving social support, such as intergenerational support, tend to have more optimistic perceptions and expectancies for their life.

On the other hand, optimism is also associated with individuals’ well-being and health status. Recent studies have demonstrated that optimism plays a significant role in predicting several aspects of subjective well-being in old age [53,54,55]. There is plenty of evidence to show that optimism has many behavioral implications when it comes to coping with difficulties, which means optimists tend to be more engaged as well as adopt more problem-focused coping strategies and approaches to regulating emotions, all contributing to better functioning [23,26]. In the context of aging, which is often associated with losses and challenges, optimism is shown to be a psychologically beneficial resource that can assist the elderly with coping with these difficulties, preventing depression, as well as conferring psychological benefits [56].

Taken together, the mediating effect of optimism on the association between received intergenerational support and subjective well-being has been identified in the present study. This is in line with previous literature that shows that optimism mediates the relationships between social support and related well-being outcomes [28,57]. One plausible explanation draws from the relational regulation theory proposed by Lakey and Orehek [58], which demonstrates the influencing mechanisms underlying the impact of social support on positive psychological well-being. As suggested, social support provides opportunities for daily social interactions that successfully regulate the emotions and actions of individuals, which in turn assist with affirming psychological well-being [59]. Optimism represents the positive thoughts and feelings about oneself that can be formulated by intergenerational support, further maintaining older parents’ emotional well-being. When an elderly person receives more intergenerational support, their evaluation of the present and their expectations for the future are more positive, which in turn, results in a higher level of happiness. To the best of our knowledge, this study is the first one to prove the indirect effect of intergenerational support on subjective well-being through the mediating role of optimism.

### 4.3. Sex Differences

Beyond our expectations, there is no significant difference existing between male and female groups in the theoretical model as indicated by the multigroup comparison. Although the findings contradict previous literature that reported a discrepancy between male and female seniors in aspects of the association between intergenerational support and subjective well-being [60], the present study echoes the sex similarities hypothesis proposed by Hyde [61]. As suggested, males and females are more alike than different in most psychological traits and abilities. According to these findings, even if the prevalence of received intergenerational support, optimistic beliefs, and the happiness of male and female elders differed, there were no significant differences in the interrelationships between these variables.

### 4.4. Limitations and Implications

The present study still has some limitations that should be resolved in the future. First, we applied cross-sectional data from CGSS 2017, so that the direct effects of intergenerational support on subjective well-being and the indirect effects through optimism should be interpreted with caution. Future studies should adopt longitudinal data to build causal relationships among the variables. Second, this study only examined the influencing role of the intergenerational support received from adult children by elderly parents, ignoring the potential effects of providing intergenerational support on their optimism and subjective well-being. As found in the literature, scholars believe that elders who provide intergenerational financial/instrumental/emotional support are likely to have better well-being due to an enhanced self-perception. However, it remains unclear how optimism could be the intermediary mechanism between providing intergenerational support and subjective well-being. Third, the findings of the current study cannot be generalized to the whole population of elderly Chinese. Since our participants were those elders who have at least one adult child and may have opportunities to receive intergenerational support from them, other elders who are childless and who lack intergenerational support from adult children have been excluded. Future studies should pay more attention to these elders and examine the psychosocial determinants for improving their subjective well-being through other types of social support. Finally, the reliabilities of the measures for intergenerational support and optimism were relatively low, and only one item was used to measure older parents’ subjective well-being. Improvements should be made in future research by using more reliable items to examine the variables, which may increase the research validity.

Despite these limitations, the present study has contributed to the development of knowledge by providing evidence to support the claim that intergenerational support from adult children has a direct effect on the subjective well-being of the elderly in a Chinese context, as well as an indirect effect through optimism. Our findings advance Karademas’s [26] model by introducing the proposed casual chain of social support–optimism–subjective well-being into the context of intergenerational interactions, with the focus on the support from the younger generation to the older generation. It further highlights that the elderly’s subjective well-being is jointly affected by external resources they can attain (e.g., intergenerational support) and internal resources from their personality characteristics (e.g., optimism).

The results of this study also enrich our practical understanding of healthy aging. It was noted that intergenerational support received from children, particularly instrumental and emotional support, and optimism of the elderly about their lives were protective factors for maintaining elderly people’s subjective well-being. Therefore, the government should encourage children to perform supportive filial behaviors for their aging parents through policymaking and public education. The fine tradition in Chinese culture—filial piety that encourages children to love, respect, and care for their older parents—should be carried forward. Additionally, service providers could design interventions using positive psychology for improving the elderly’s optimistic beliefs about life to achieve healthy aging. For example, the Best Possible Self intervention, one of the most widely applied projects promoting a positive view of oneself in the best possible future [62], could be redesigned and indigenized for Chinese elders in the future. Moreover, because of the sex similarity found in the present study, these interventions could be applied to both male and female elders.

## Figures and Tables

**Table 1:** Means and standard deviations of each scale by sex group (standard deviations in parenthesis).

	Overall	Sex Groups	
		Male	Female
Received intergenerational support	8.61	8.38	8.81
	(2.64)	(2.56)	(2.68)
Received financial support	2.91	2.85	2.96
	(1.18)	(1.15)	(1.21)
Received instrumental support	2.77	2.71	2.82
	(1.18)	(1.16)	(1.21)
Received emotional support	2.93	2.82	3.03
	(1.02)	(1.00)	(1.03)
Optimism	11.43	11.47	11.39
	(1.81)	(1.77)	(1.84)
Subjective well-being	3.90	3.88	3.91
	(0.85)	(0.88)	(0.83)

**Table 2:** Intercorrelations between variables in the theoretical model.

	1	2	3	4	5
1. Received financial support	1	0.39 **	0.34 **	0.01	0.05
2. Received instrumental support		1	0.49 **	0.06 *	0.11 **
3. Received emotional support			1	0.14 **	0.13 **
4. Optimism				1	0.37 **
5. Subjective well-being					1

Note. ** *p* < 0.01; * *p* < 0.05.

## Data Availability

Publicly available datasets were analyzed in this study. The Chinese General Social Survey 2017 data can be found here: http://www.cnsda.org (accessed on 23 July 2021).
